# The Impact of Different Fiber Placement Techniques on the Fracture Resistance of Premolars Restored with Direct Resin Composite, In Vitro Study

**DOI:** 10.3390/jfb16060225

**Published:** 2025-06-17

**Authors:** Reham Hesham Ibrahim, Dina Wafik ElKassas, Sameh Mahmoud Nabih, Mennatallah Naguib Salem, Rasha Haridy

**Affiliations:** 1 Conservative Dentistry Department (Restorative Division), Faculty of Oral and Dental Medicine, Misr International University, Cairo 11785, Egypt; mennatallah.salem@miuegypt.edu.eg; 2 Department of Clinical Dental Sciences, College of Dentistry, Princess Nourah bint Abdulrahman University, P.O. Box 84428, Riyadh 11671, Saudi Arabia; dwelkassas@pnu.edu.sa; 3 Restorative Dentistry Department, Faculty of Dental Medicine (Boys, Cairo), Al-Azhar University, Cairo 11361, Egypt; samehmnabih@gmail.com

**Keywords:** fiber-reinforced composite, fracture resistance, polyethylene, occlusal splinting, wallpapering

## Abstract

Fiber-reinforced composites (FRCs) are recognized for enhancing the fracture resistance of structurally compromised teeth. However, the optimal orientation and placement of fibers in direct resin composite restorations remain under debate. This study aimed to evaluate the fracture resistance of maxillary premolars with mesio-occluso-distal (MOD) cavities restored using polyethylene fibers with different placement techniques, compared to conventional incremental composite restoration. Methods: Sixty intact maxillary premolars were randomly assigned to six groups (n = 10). Group 1: intact teeth (positive control); Group 2: MOD cavity without restoration (negative control); Group 3: MOD cavity restored with nanohybrid composite using the incremental technique; Group 4: polyethylene fiber placed on the pulpal floor; Group 5: fiber placed circumferentially along cavity walls (wall-papering technique); Group 6: fiber placed buccolingually in an occlusal groove (occlusal splinting). Fracture resistance was assessed using a universal testing machine. Failure mode was also analyzed. Results: Group 6 (occlusal splinting) exhibited the highest fracture resistance (1137.72 ± 316.20 N), significantly exceeding Group 3 (546.93 ± 59.89 N) and other fiber-reinforced groups (*p* < 0.05). Failure mode analysis revealed no significant differences between the fiber-reinforced groups and the intact teeth. Group 6 also had the highest percentage of restorable fractures (90%). Conclusions: Incorporating polyethylene fibers, especially through occlusal splinting, significantly improves fracture resistance in MOD-restored maxillary premolars. This technique may offer a promising alternative to conventional composite restorations in structurally weakened posterior teeth.

## 1. Introduction

The use of resin composites in restorative dentistry has gained significant popularity due to their aesthetic properties, durable bonding—achieved through micromechanical retention and adhesive systems—and their compatibility with dental tissues [1,2]. Despite these advantages, composite resins are inherently brittle and exhibit limited fracture resistance, defined as a material’s capacity to absorb energy and resist crack propagation [3]. Another major limitation of direct composite restorations is polymerization shrinkage. This phenomenon can induce internal stresses within the material, leading to microleakage, secondary caries, formation of microcracks, and eventual restoration failure [3,4,5,6]. Although incremental layering techniques have been proposed to mitigate these effects, challenges persist in restoring teeth with extensive structural loss, particularly in the posterior region. Maxillary premolars are especially susceptible to vertical fractures, even more so than molars, despite the latter experiencing higher masticatory forces [7]. This vulnerability is attributed to their anatomical configuration and the concentration of occlusal loads, which predispose them to cusp fractures. Consequently, restoring severely compromised premolars with conventional composite techniques may not always yield optimal outcomes [8,9].

To address these limitations, fiber-reinforced composites (FRCs) have been introduced as a viable reinforcement strategy. These materials aim to enhance the fracture resistance of restored teeth by distributing functional stresses more effectively and preventing crack propagation [10,11,12]. FRCs are available in various forms and can be classified based on material composition, fiber architecture, surface impregnation, and their intended use, whether chairside or in a laboratory setting. Carbon, glass, and polyethylene fibers are the most common types of fibers used.

Short-fiber-reinforced composites (SFRCs) consist of fibers which are discontinuous, oriented randomly, and pre-integrated into the resin matrix by the manufacturer. In contrast, long fiber-reinforced composites—also known as continuous fiber composites—are manually integrated by the dentist during restoration fabrication. Long fibers may be arranged in a unidirectional manner, parallel to each other, or in multidirectional woven or mesh patterns [12,13,14,15]. Key factors influencing FRC performance include fiber density, surface treatment (which affects fiber–resin bond efficacy), fiber orientation relative to the loading direction, and fiber positioning within the restoration [13,16,17].

A number of studies have demonstrated that incorporating high-molecular-weight polyethylene fibers into large composite restorations improves load distribution by acting as stress breakers and effectively enhances the fracture strength of compromised vital and non-vital teeth [18,19,20]. Although the beneficial effects of these fibers are generally recognized [21,22], there remains ongoing debate regarding the optimal orientation and placement technique within the restoration. Despite numerous fiber configurations being explored, no definitive guidelines or consensus currently exist to guide clinicians in selecting the most effective technique to maximize mechanical benefit.

In clinical practice, deep and extensive MOD cavities in vital premolars are a common and challenging scenario, often resulting from caries or trauma. Identifying effective reinforcement strategies for these structurally compromised teeth is, therefore, of high clinical importance. The aim of this study was to assess and compare the reinforcing effects of different polyethylene fiber placement techniques regarding the fracture resistance of extensive cavity preparations in maxillary premolars. This is the first study to our awareness that evaluates and compares three clinically relevant polyethylene fiber placement techniques in vital maxillary premolars with standardized deep MOD cavities. By analyzing both fracture resistance and failure patterns in a controlled in vitro setting, this research provides clinically applicable insights to support evidence-based decision making in restorative dentistry. The null hypothesis stated that there would be no effect on the fracture resistance of maxillary premolars with MOD cavity preparations restored using polyethylene fibers with different placement techniques or incremental packing of direct resin composite.

## 2. Materials and Methods

The Institutional Review Board at Misr International University granted ethical approval for this study (IRB No.: MIU-IRB-2223-175). The research was conducted between March and July 2024. Sixty maxillary premolars were collected from the MIU tooth bank following extraction due to orthodontic or periodontal reasons. Sound maxillary premolars without visible caries or cracks, as assessed by magnification loupes (2.5×), and without prior endodontic treatment, crown, or root resorptions were the inclusion criteria. The maximum buccolingual dimension, mesiodistal dimension, intercuspal distance, and occlusogingival height of each tooth were measured using a digital caliper (Tooleye, Frankfurt, Germany). Teeth with similar dimensions were chosen, with an average buccolingual width of 10 millimeters (mm), mesiodistal width of 7 mm, intercuspal distance of 6 mm, and occlusogingival height of 9 mm. To maintain uniformity within and between groups, the tooth dimensions were standardized to ensure they did not vary by more than 10% from the group mean [23,24,25]. A randomization list created by www.random.org (accessed on 10 March 2024) was used to assign the chosen teeth to one of the six treatment groups (n = 10).

### 2.1. Cavity Preparation

**Figure 1:**
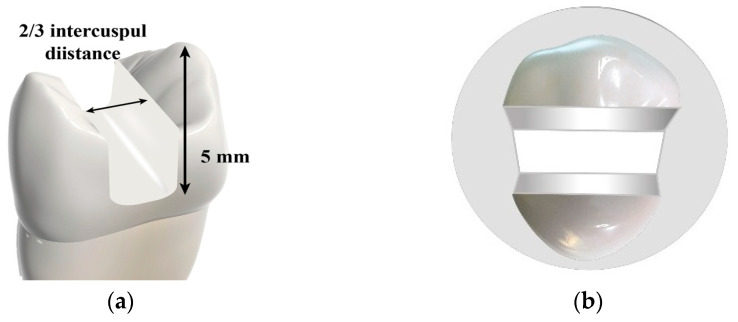
Illustrative diagram showing: (**a**) cavity dimensions; and (**b**) cavity form.

**Figure 2:**
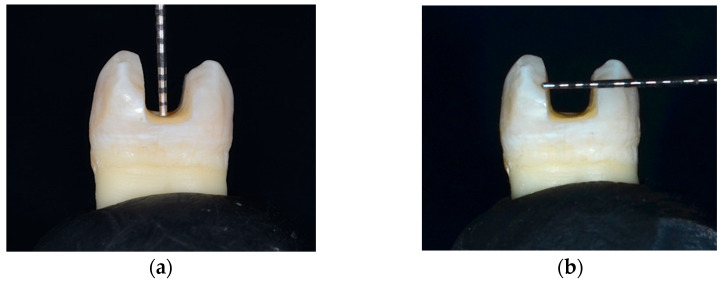
Cavity dimensions: (**a**) depth; and (**b**) width.

Deep and wide MOD cavities in maxillary premolars were prepared in five of the six groups by a single operator to simulate extensive cavities. To standardize the cavity dimensions, a digital caliper (Tooleye, Germany), and a 15 UNC graduated periodontal probe (Hu-Friedy, Chicago, IL, USA) were used. The outline was positioned at the occlusal surface’s midline, centered between the inter-cuspal distance. A round-end parallel diamond bur (881.31.014 FG-Brasseler Dent, Savannah, GA, USA) was used in a high-speed handpiece (NSK PanaAir FX, Kanuma, Japan) to prepare the cavity. The diamond bur was replaced with a new one after every five preparations. A digital caliper was used to continuously measure the thickness of the walls at the cavity base [24]. The cavity dimensions were standardized with a depth of 5 mm [26,27] and a width of 2/3 the inter-cuspal distance (approximately 4 mm) [28]. The walls of the cavity were parallel to the tooth’s long axis. The depth was determined using the 15 UNC graduated periodontal probe, measured from the pulpal floor to the buccal cusp tip. The cavity was prepared as a single continuous unit, maintaining uniform width and depth in the occlusal and proximal portions. The cavosurface margins were kept perpendicular to the unprepared tooth surface. Finally, the cavities were rinsed and air-dried [24,29,30] (Figure 1 and Figure 2).

### 2.2. Restorative Procedures

Group (1): Sound premolars left intact to serve as positive control.

Group (2): MOD prepared cavities with no restoration to serve as negative control.

**Figure 3:**
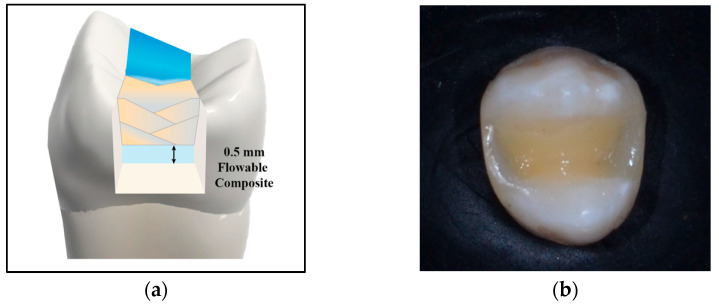
(**a**) Diagrammatic and (**b**) Photographic representation of nanohybrid composite, oblique incremental application technique.

Groups (3–6): Underwent standardized MOD cavity preparations, adhesive protocols, and initial composite layering before experimental restorative techniques were applied. The enamel was acid-etched selectively with phosphoric acid (37%) etching gel (Meta Etchant, MetaBiomed, Chungcheongbuk-do, Republic of Korea) for 15 s. The acid was then rinsed with an air water spray for 20 s and blot dried with a cotton pellet following manufacturer instructions. A disposable microbrush was used to apply a universal adhesive (3M^TM^ Single Bond Universal, 3M ESPE, Neuss, Germany) to the prepared cavity and rubbed in for 20 s. This was followed by a gentle air stream for approximately five seconds to allow evaporation of the solvent completely. The adhesive layer was then light-cured for ten seconds, according to manufacturer instructions, using an LED light cure device (LED F, Woodpecker Instruments Co., Guilin, China) with 1600–1800 mW/cm^2^ intensity. A built-in radiometer was used to check and verify the light intensity after every ten uses [1]. A Tofflemire metallic matrix band was placed. First, a layer of flowable composite of approximately 0.5 mm thickness (3M^TM^ Filtek^TM^ Supreme Flowable Restorative, 3M ESPE, Maplewood, MN, USA) was applied to the pulpal floor of the cavity and light-cured for 20 s according to manufacturer instructions [24]. Then, the interproximal walls were built, with a thickness of approximately 1 mm, up to the level of the marginal ridge using a plastic application instrument (Condensa LM-Arte^TM^, Pargas, Finland) and nanohybrid composite resin (Filtek™ Z250 XT Universal Restorative, 3M ESPE, Maplewood MN, USA). Following this closed centripetal technique, the MOD cavity was converted into a class I cavity [23]. The remaining cavities were then restored according to the different direct restorative techniques as follows:

Group (3): An oblique incremental application technique with nanohybrid composite restorative material was used to restore the cavity. Increments were cured from the occlusal surface while holding the light guide tip closely to the restoration. The exposure time was 20 s for each increment, and maximum increment thickness was 2 mm following manufacturer instructions. Finishing and polishing of the restorations was performed using fine diamond burs and diamond impregnated polishers (Eve DiaComp Twist, Keltern, Germany) (Figure 3).

**Figure 4:**
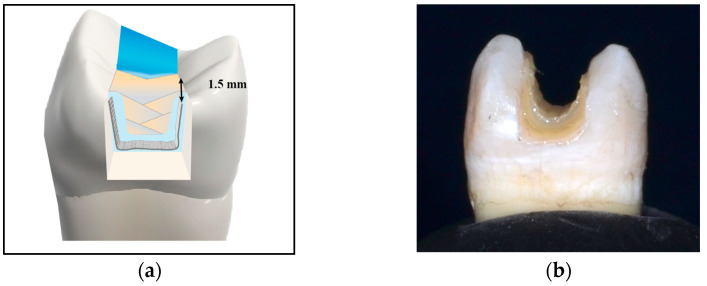
(**a**) Diagrammatic and (**b**) Photographic representation of the pulpal floor application technique.

Group (4): A leno woven ultra-high-molecular-weight polyethylene ribbon fiber (Ribbond-Ultra; Ribbond Inc., Seattle, WA, USA), 3 mm in width, was removed from the package using cotton pliers. After determining the required length with tin foil according to manufacturer instructions, the fiber piece was cut, using the specific scissors included in the Ribbond kit to ensure a sharp clean cut. Prior to application within the cavity, the fiber was wetted with an unfilled resin (Ribbond wetting resin, Ribbond Inc, Seattle, WA, USA). Using a plastic instrument parallel to the fiber’s length, the excess resin was squeezed out of the fiber [31]. A layer of flowable resin composite was applied to the cavity surfaces. The fiber was then embedded within the layer of uncured flowable composite, inserted horizontally on the pulpal floor of the cavity in a buccolingual direction (U-shaped). The fiber was adapted closely, using a plastic instrument, to the pulpal floor, and buccal and lingual walls of the cavities, extending from the occlusal third of the internal buccal wall to the occlusal third of the internal lingual wall, making sure the fiber did not reach the cavity margins. Approximately 1.5 mm of space was left for the final composite layer occlusally. After light curing for 20 s, the rest of the cavity was restored with nanohybrid composite as described before using the incremental technique, in which each increment was cured for 20 s [23,24,32] (Figure 4).

**Figure 5:**
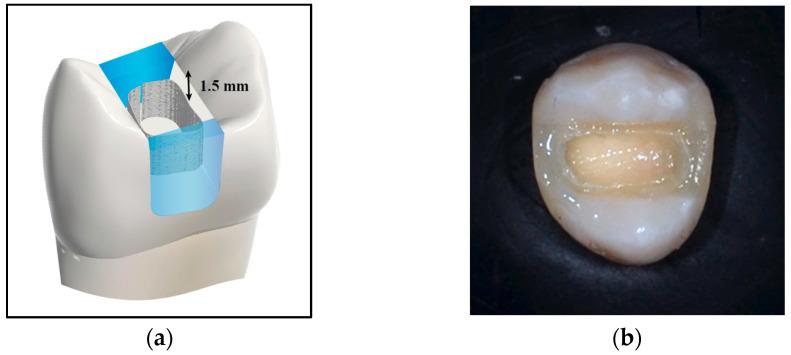
(**a**) Diagrammatic and (**b**) Photographic representation of the circumferential, wallpapering application technique.

Group (5): Ribbond fiber was applied in a circumferential, wallpapering technique as described by Deliperi et al. [31] and Sáry et al. [24]. After buildup of the proximal walls as described, a piece of fiber was cut to the required length. The fibers edges were sharp. The fiber was wetted with unfilled resin as described in Group 4. A coat of flowable composite was applied circumferentially to the vertical walls of the cavity (<1 mm in thickness) [1]. The fiber piece was placed vertically into the layer of uncured flowable composite and adapted closely to the inside of the buccal, proximal, and palatal walls. The Ribbond edges overlapped one another, folding down onto the axio-pulpal line angles of the cavity. The fiber ended 1.5 mm below the cavosurface margin [23,24,31]. Curing was performed for 20 s followed by restoration of the cavity with nanohybrid composite as described in Group 3 (Figure 5).

**Figure 6:**
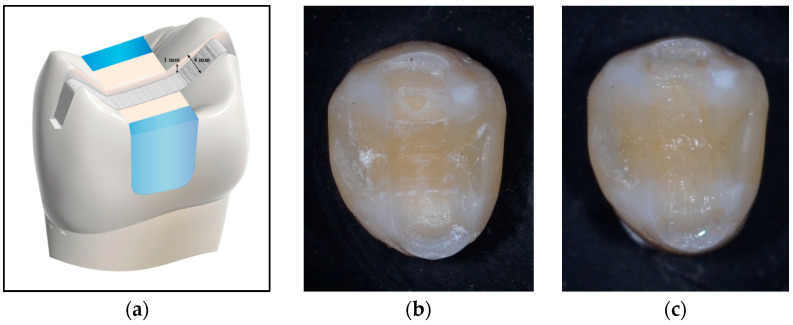
(**a**) Diagrammatic and Photographic representation of (**b**) the occlusal groove preparation and (**c**) application technique.

Group (6): The MOD cavity was first restored completely with the nanohybrid composite incremental technique as described previously in Group 3. A groove 4 mm wide and 1 mm deep was prepared on the occlusal surface of the finished restoration, using a high-speed diamond bur. The ends of the groove started at the occlusal 1/3 of the external buccal surface, above the maximum contour, extended between the cusp tips, and ended at the occlusal 1/3 of the external lingual surface, above the maximum contour. Enamel etching and adhesive treatment of the groove were performed as described previously. Flowable resin was used to line the groove, and a prewetted piece of three-millimeter-wide polyethylene fiber was embedded into the uncured resin inside the prepared groove. After adaptation and curing of the fibers for 20 s, the cavity was restored with a final occlusal layer of nanohybrid composite to cover the exposed fibers and restore occlusal anatomy [18,24,33,34] (Figure 6).

### 2.3. Thermocycling Procedure

Thermal cycling treatment was performed for all the specimens for 5000 cycles. The temperatures were set between 5 °C and 55 °C. Each cycle included a dwell time of 20 s and a transfer time of 5 s, effectively simulating six months of aging in a clinical environment [35,36,37]. These parameters were selected according to the thermal cycling protocols suggested over the past two decades by the International Standards Organization (ISO) (1994) and by Gale and Darvell [38], who estimated that roughly 10,000 cycles are equivalent to one year of clinical function [38,39].

### 2.4. Embedding of Teeth

To allow for simulation of the periodontal ligament and alveolar bone, the root surfaces were dipped in melted setup wax (Cavex Holland B.V, Haarlem, The Netherlands), forming a coat with a uniform layer of approximately 0.3 mm thick. The teeth were then vertically embedded approximately 2 mm apical to the cemento-enamel junction (CEJ) in self-cured acrylic resin (Acrostone™, Cairo, Egypt) housed within cylindrical molds made of poly vinyl chloride (PVC) pipes (diameter 2 cm, height 2 cm) to simulate the alveolar bone level. After setting of the self-cured acrylic resin, the wax was eliminated by placing in warm water. The space created between the root and the resin block was filled with light body poly-vinyl siloxane impression material (Speedex, Coltene Whaldent, Altstätten, Switzerland) to mimic the periodontal ligament. Excess impression material was trimmed using a surgical blade [24,33,40,41]. The teeth were numbered at the bottom of the resin block according to the sample grouping to be prepared for mechanical testing.

### 2.5. Fracture Resistance Test

A universal testing machine (Instron 3343, Norwood, MA, USA) was used. A 4 mm steel sphere was used to apply an axial compressive load to the mounted specimens [4,33,42]. The applied crosshead speed was 1 mm min^−1^ [36,43]. The sphere was positioned over the center of the tooth, aligned parallel to the tooth’s long axis, until it made simultaneous contact with the restoration’s occlusal surface and the buccal and lingual cusp inclinations. Load was applied until a fracture occurred, and for each specimen, a force versus extension curve was generated. The fracture threshold was determined as the initial load responsible for the fracture, recorded in Newtons (N) [36,43].

### 2.6. Failure Mode Analysis

Fractured specimens were visually examined using magnification loupes (2.5×), to examine failure modes (the type and location of failure). Fractures were classified as restorable if the fracture line was located above the CEJ or within one millimeter or less apical to it; and they were considered non-restorable if the fracture line extended apically by more than one millimeter to the CEJ [44,45,46].

### 2.7. Statistical Analysis

Numerical data from the experiment was collected and tabulated. Normality of the data was analyzed using the Kolmogorov–Smirnov and Shapiro–Wilk tests. The fracture resistance values followed a normal (parametric) distribution and were expressed as mean ± standard deviation. One-way ANOVA was applied to compare differences among the groups and a post hoc test was used to detect significance if present. Data on failure modes was displayed as percentages and frequencies. The failure modes of different groups were compared using Fisher’s exact test. A significance level of *p* ≤ 0.05 was set. Version 23.0 of IBM SPSS statistics for Windows, IBM Corp., Armonk, NY, USA, was utilized for statistical analysis.

## 3. Results

### 3.1. Fracture Resistance (N)

Data in Table 1 shows the results of the one-way ANOVA test for comparison of the fracture resistance load for the different restorative treatments. Results of the one-way ANOVA revealed a statistically significant effect of the different restorative treatments on the fracture resistance load (*p* < 0.0001).

**Figure 7:**
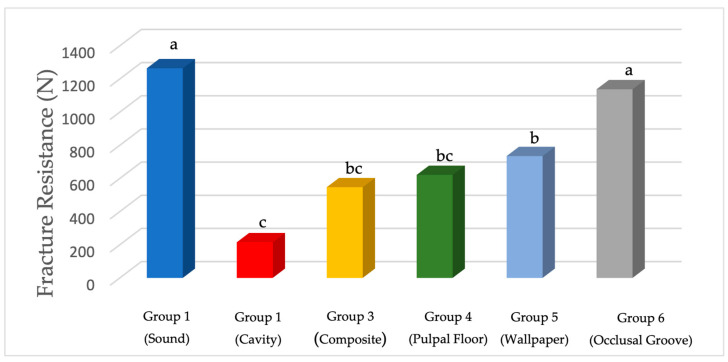
The mean fracture resistance values in Newtons (N). Lower-case letters indicate significance between tested groups.

The results of the study indicated significant differences in fracture resistance among restorative treatments. Group 1 (positive control) had the highest average fracture resistance, while Group 2 (negative control) had the lowest at 214.87 ± 34.49 N. Among the restored groups, fiber occlusal splinting (Group 6) showed the highest fracture resistance, similar to Group 1, whereas Group 3 (direct composite resin) had the lowest resistance. Groups 4 (pulpal floor) and 5 (wallpapering) displayed moderate resistance increases but were not significantly different from Group 3 (Table 2) (Figure 7).

### 3.2. Failure Mode Results

**Figure 8:**
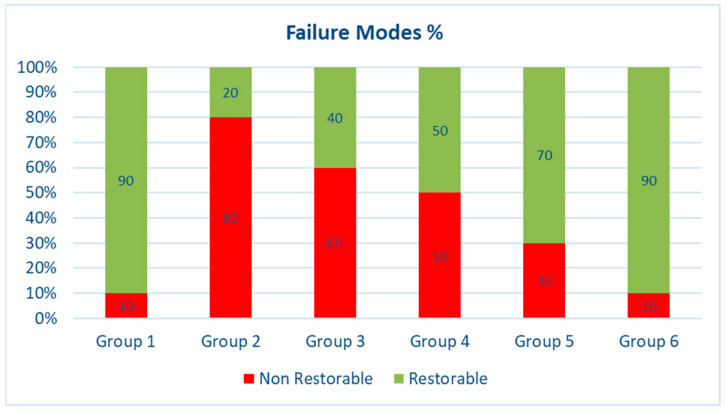
Failure modes (%) of the different groups used in the study (Group 1, sound; Group 2, cavity; Group 3, composite; Group 4, pulpal floor; Group 5, wallpaper; Group 6, occlusal groove.

**Figure 9:**
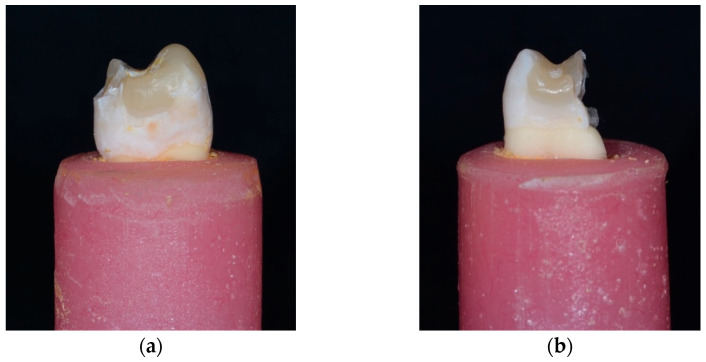
Failure mode: (**a**) Restorable and (**b**) Non-Restorable.

In terms of failure modes, fracture patterns were classified as either restorable or non-restorable, based on the criteria described by Scotti et al. [45]. A fracture was considered restorable if it occurred above or up to 1 mm apical to the CEJ, indicating that the tooth could be restored using conventional techniques. In contrast, a non-restorable fracture extended more than 1 mm below the CEJ, typically requiring extraction. Group 6 exhibited the highest rate of restorable failures (90%), demonstrating its effectiveness in reducing severe, non-restorable fractures. The failure types observed in each group are summarized in Table 3, and representative examples are shown in Figure 8 and Figure 9.

## 4. Discussion

Fiber reinforcement of dental composites offers a minimally invasive strategy to enhance the toughness and fracture resistance of direct restorations. This approach aligns with the principles of biomimetic restorative dentistry, which aims to restore tooth function and durability using materials and techniques that mimic natural tissue. In this context, fiber reinforcement is considered biomimetic because it seeks to emulate the mechanical behavior of dentin, particularly its ability to resist crack propagation, thereby strengthening both the restoration and the structurally compromised tooth [11,12,22,47].

Ribbond is a type of long fiber-reinforced ribbon made from a leno woven ultra-high-molecular-weight (LWUHMW) polyethylene fiber. This material’s woven fabric is composed of cross-linked fine polyethylene strands. It is flexible and demonstrates high tensile strength, modulus of elasticity, and fracture toughness [27]. The fiber is treated with cold gas plasma to improve adhesion to synthetic restorative materials, including composite resins. Its distinctive fiber structure facilitates effective force distribution, while its flexibility allows it to conform easily to the shape and contours of cavity walls. Polyethylene fibers provide a layer of stress absorption that helps redirect cracks and fractures and serve to splint the tooth internally, thereby increasing its fracture strength [20]. Furthermore, its translucency enhances its aesthetic qualities, resulting in its extensive use in dental restorations with composite resin materials [24,48].

Composites are typically isotropic, displaying similar properties in all directions. However, incorporating fibers changes their behavior from isotropic to non-isotropic materials, leading to modified mechanical performance based on the direction of applied forces [24,33]. Long continuous fibers provide anisotropic mechanical properties, meaning their efficacy can be further enhanced when utilized in different orientations, which improves fracture resistance [24,49]. Various techniques have been investigated. Nevertheless, the optimal application techniques to maximize the mechanical performance of severely damaged teeth remain unclear. Therefore, this study was carried out to clarify the impact of different applications/orientations of polyethylene fibers on the fracture resistance of directly restored structurally compromised premolars. The results of the present study revealed a statistically significant difference in the fracture resistance between groups restored with different fiber orientations. Thus, the null hypothesis was rejected.

The intact teeth in the positive control group (Group 1) exhibited the highest mean fracture resistance values, while the unrestored teeth in the negative control group (Group 2) demonstrated the lowest and were associated with the highest percentage of non-restorable failure (80% catastrophic failure). These findings are consistent with previous research [8,9,50]. It is suggested that exerting a load on unrestored teeth causes a wedge effect to arise between the buccal and palatal cusps, resulting in diminished fracture resistance and severe fractures [51]. Moreover, the significant weakening of posterior teeth during MOD cavity preparations can lead to increased stress on cavity walls, primarily influenced by the depth of the cavity and the absence of marginal ridges. Larger cavity dimensions result in greater cuspal deflection [25]. Hood [52] noted that the cavity floor serves as a fulcrum for cusp bending, with the length of the cantilever cusp wall increasing as the depth of the cavity increases, a finding also supported by Forster et al. [29].

Group 3, which consisted of teeth restored solely with direct composite resin, exhibited the lowest mean fracture resistance among the restored groups with a statistically significant difference when compared to Group 6 that received Ribbond reinforcement applied as occlusal splinting. This was in agreement with Albar and Khayat [2], who also compared non-reinforced conventional nanohybrid class II composite restorations to fiber reinforcement using Ribbond and found a significant difference. The authors explained this from a mechanical perspective. During application of a compressive load, such as the axial compressive load employed in this research, complex stresses develop within the material. The compressive forces are converted into shear and tensile forces, which are transmitted in a transverse direction to the cavity walls and floor, potentially initiating cracks. Due to the composite resin’s inherent lack of toughness, the propagation of these cracks occurs more freely, resulting in catastrophic failure [2,23]. Failure mode analysis further supported this conclusion, revealing that 60% of the samples in Group 3 experienced non-restorable fractures, highlighting the suboptimal fracture toughness of composite resin.

When compared to non-reinforced restorations, the mean fracture resistance of Groups 4, 5, and 6 that were reinforced with Ribbond fibers was higher, yet the increase was statistically significant only when fibers were applied in the occlusal splinting technique (Group 6). Group 4, which involved polyethylene fibers applied on the pulpal floor of the cavity, connecting the opposing buccal and palatal walls, showed the weakest fracture resistance among the polyethylene fiber groups with no significant difference between the direct composite resin group or the circumferential wallpapering group. This was in agreement with Sáry et al. [24], who studied the reinforcing effect of various direct restorative placement techniques using Ribbond in MOD cavities of mandibular third molars. This result was also in agreement with Akman et al. [33], who directly compared three placement techniques of Ribbond, on the pulpal floor, circumferentially, and through an occlusal groove, to reinforce endodontically treated (ETT) mandibular first molars. Both previous studies found that the effectiveness of Ribbond fibers was irrespective of their position inside the restoration, as there were no significant differences in fracture resistance among the fiber positions as well as the conventional non-reinforced composite group. The diminished fracture resistance observed in Group 4 (pulpal floor) could be attributed to the inherent mechanical properties of the polyethylene fibers when placed in this configuration. Although the walls of the restoration were connected by the fibers, the absence of tension across the fibers may have limited their ability to distribute stress effectively throughout the restoration. Research has indicated that positioning of fibers on the tensile side of a restoration can significantly enhance the flexural properties of the composite resin. Consequently, when fibers are not subjected to tensile forces, their reinforcing potential may not be fully reached [17,24,33].

In partial contradiction to our study, Abdulamir and Majeed [23] evaluated the effects of different fibers: glass fiber post, SFRC, and Ribbond fibers placed on the pulpal floor as well as in a circumferential technique, on the fracture of ETT maxillary premolars. They noted a significant difference in both Ribbond groups compared to composite resin alone, however, no difference was seen between the two placement techniques. They explained that when Ribbond is applied to the cavity floor, it is intended to function as a crack stopper by redirecting stress and dissipating strain. This stress-modifying effect enhances the overall toughness of the restoration [23]. This contradiction could be due to the different size of the Ribbond fiber used in their study which was 4 mm wide compared to the 3 mm wide fiber used in this study, as well as the different type of composite material used. Ayad et al. [53] showed contradicting results when investigating the effect of Ribbond reinforcement on the fracture strength of weakened marginal ridges in molar teeth. Class I and compound class II cavities restored with conventional composite were compared to restorations reinforced with Ribbond applied to the pulpal floor. Reinforcement with Ribbond resulted in significantly higher fracture resistance. The differences in the results could be explained by the different testing conditions. The authors utilized different cavity designs in molars, which affect load distribution on the remaining tooth structure. Furthermore, a 5 mm stainless steel bar was centered over the marginal ridge in their study, unlike this study where MOD cavities were prepared in premolars, and the load was centered over the occlusal surface, between the buccal and lingual cusps, using a 4 mm stainless steel ball.

Another fiber reinforcement application technique involves the circumferential application of fiber to the inner cavity wall surfaces, commonly referred to as the wallpapering technique [31]. In the present study, the wallpapering technique (Group 5) yielded high mean fracture resistance values, statistically significant from the negative control group (Group 2), yet statistically similar to the direct composite group (Group 3) and the pulpal floor group (Group 4). Additionally, the wallpapering group showed statistically enhanced failure modes with 70% of the samples showing restorable fractures. Other studies have shown that Ribbond fibers can enhance the fracture resistance of direct composite restorations when applied using this technique [23,24,26].

In partial agreement with our study, Gómez et al. [26] compared the effect of fibers placed on the pulpal floor and circumferential (around the axial walls) on the fracture resistance of ETT premolars. The results indicated both patterns exhibited significantly higher fracture load values when compared to the composite control group without fiber, however, no significant difference was found between the two patterns. The results of Abdulamir and Majeed [23] were also in partial agreement with the results of our study. The authors evaluated the wallpapering technique as mentioned previously and showed improved fracture resistance compared to conventional composite. This improvement may be due to the Ribbond fibers’ ability to mimic the dentino-enamel complex when placed closely against the cavity walls, reinforcing the crack shielding mechanism and allowing the tooth structure and composite materials to work together harmoniously under strain.

Furthermore, properly adapting and curing the Ribbond fibers to the contours of the remaining tooth structure helps reduce the amount of composite resin between the fiber and the tooth, thereby protecting the remaining walls from stresses caused by occlusal loads and polymerization shrinkage and minimizing the formation of defects and voids that could lead to crack propagation and microleakage [1,8,23,26].

Although the results of our study showed improvement in fracture resistance with the wallpapering technique, the results were not statistically significant compared to the conventional composite group (Group 3). This outcome is consistent with findings from Hazar and Hazar [54]. This could be due to the challenge faced during the adaptation of the fibers. The authors suggested that the reduction in fracture strength observed with this fiber placement technique may be influenced by the shape memory characteristics of the material. This property complicates the application of fibers using the circumferential and pulpal floor techniques, particularly in narrow cavities where bending is required for proper adaptation. Such bending can result in inadequate adhesion, leading to voids and incomplete polymerization, which ultimately affects the performance of the restoration. In clinical settings, adapting this material to fit narrow cavities, especially in posterior areas and premolars where space is limited, poses significant challenges, potentially limiting the effectiveness of these techniques in practice. It is important to note that the authors employed glass fibers in their research, which possess different properties compared to polyethylene fibers. These differences may complicate direct comparisons between the two studies, as the mechanical behavior of glass fibers may differ from those of polyethylene fibers used in direct composite restorations [54].

In the present study, fibers applied as occlusal splinting (Group 6), exhibited the highest mean fracture resistance with a statistically significant difference compared to all the restored groups. Fracture resistance increased significantly, reaching statistically similar values to the intact group. Additionally, this group exhibited the most favorable failure outcomes compared to the other groups, highlighting the superior performance of this technique. The results of this study are consistent with Oskoee et al. [55], who studied the impact of three fiber placement techniques on the fracture resistance of maxillary premolars. In their study, glass fibers were positioned in the gingival, middle, and occlusal thirds of the cavity. They found that fracture resistance was significantly greater in the group with occlusal fiber placement compared to the other groups. The fiber position, which is extended through a groove prepared on the occlusal third of the buccal and lingual walls, allows the fibers to be placed near the force application point, resulting in a shorter working arm according to the lever’s principle, which increases fracture resistance. Additionally, the fibers help keep the buccal and lingual cusps together, effectively stabilizing the cusps and further contributing to their strength [55]. The results were also in agreement with Küçük and Keçeci [56], who evaluated the effect of five fibers with various placement designs in root-canal-treated and bleached premolars. Intracanal, intracoronal, and intercuspal (occlusal splinting) fiber placement designs were compared. Fibers placed as occlusal splinting exhibited a significantly better strengthening effect compared to the other groups. This could be attributed to the fact that, when polyethylene fibers are placed in the occlusal third of the crown, they are expected to provide additional benefits. This includes acting as an early stress-redirecting layer. Biomechanically, the dense network of locked nodal intersections in polyethylene fibers may also function as an early crack-stopping mechanism, potentially serving as a substitute for the dentin–enamel junction [24,56].

A study by Agrawal et al. [8] showed results in agreement with the current study, suggesting that the enhancement in the occlusal fiber group could be due to the orientation of fibers, which significantly affects the strength of restorations. They compared different horizontal and vertical fiber orientations which were used to reinforce MOD cavities in maxillary premolars. Horizontal fiber orientation was found to provide greater fracture resistance than the vertical orientation. They attributed this to the fact that, when the longitudinal axis of the fibers is positioned in a perpendicular orientation to compressive forces, reinforcement occurs. However, when fibers are aligned parallel to compressive forces, minimum reinforcement occurs, potentially leading to matrix-dominated failures [8]. This could further explain the suboptimal results of the vertical wallpapering group (Group 5) compared to the horizontal occlusal splinting group (Group 6).

Our results, however, contradict previous findings by Sáry et al. [24] and Akman et al. [33], who demonstrated that adding polyethylene fibers in different orientations, including occlusal splinting, did not significantly increase fracture resistance in comparison to composite fillings. This discrepancy may be due to differences in study methodologies, particularly the load-to-fracture testing speed, which was set at 5 mm/min in Akman’s study, significantly exceeding the typical range of 0.5–2 mm/min. In addition, mandibular first molars (ETT) were used in their study. Sáry et al.’s study also utilized mandibular third molars. This may have affected the load distribution and subsequently the fracture load values. Bahari et al. [57] also found no enhancement in fracture resistance with long fibers for occlusal splinting compared to composite. However, in their study, a different fiber type (Interlig glass FRC) was used. This could be explained by differences in the characteristics and quantity of fibers used, as well the dimensions of the prepared occlusal groove which was only 2 mm wide and 1 mm deep.

To provide clinically relevant insight, it is important to evaluate not only the load necessary to cause fracture but also the type of fracture. Therefore, after mechanical testing, fracture patterns were classified as either restorable or non-restorable, based on the criteria described by Scotti et al. [45]. This distinction is critical, as restorations that achieve high fracture resistance but result in unfavorable (non-restorable) failure patterns may offer limited clinical value. Ideally, an effective restoration should combine mechanical strength with a favorable, restorable fracture pattern [58].

When fibers were integrated inside the direct restoration, the failure was dominantly favorable (above the CEJ). Group 6 (occlusal splinting) demonstrated the highest percentage (90%) of repairable fractures followed by Group 5 (wallpapering) at 70% and Group 4 (pulpal floor) at 50%, while Group 3 (composite alone) yielded the lowest ratio of 40% among the restored groups.

The ability to absorb stress and arrest cracks is primarily a function of the dentine–enamel junction and the surrounding dentine. However, structurally compromised teeth have a smaller dentine–enamel junction and less sound dentine, increasing the risk of catastrophic failures in the restoration–tooth complex. Using materials with high fracture toughness is beneficial as they resist crack initiation and propagation, making them suitable replacements for missing dentine–enamel junction or dentine [24,59]. Fiber-reinforced restorations provide a fail-safe mechanism where fractures occur above the cemento-enamel junction (CEJ), thus avoiding catastrophic failures and preserving the remaining tooth structure. Additionally, strategically placing the fiber against cavity walls optimizes stress distribution and energy absorption, helping to prevent failures in large cavities [8,48].

Although the results showed no significant difference regarding the fracture resistance of the group restored with composite resin alone, and those restored with Ribbond fibers in either the pulpal or wallpaper directions, the latter two groups demonstrated improved fracture modes. Furthermore, the occlusal fiber group demonstrated the most favorable failure outcomes.

These findings agree with the results of Hazar and Hazar [54], who reported that most restorable fractures were observed in a modified transfixed group in which fibers were transfixed between the buccal and palatal walls in an occlusal position among the experimental groups. Abdulamir and Majeed [23] also found that Ribbond improved fracture modes, whether applied on the cavity floor or using a “wallpapering” technique along the cavity walls, due to its ability to modify stress and create a dentino-enamel-like junction between the fibers and the tooth structure. This was attributed to the crack-stopping and crack-deflecting mechanisms of Ribbond. The three-dimensional leno weave fiber structure creates mechanical interlocking with resin, minimizing microcracking during polymerization. In contrast, unreinforced composite samples experience rapid crack propagation due to brittleness. The incorporation of fibers transforms this dynamic; they serve as crack stoppers and toughening agents by creating multiple interfaces that slow down crack growth. Minor cracks that develop are contained within areas defined by the interwoven fibers, and upon reaching the fiber plane, their progression is impeded by causing them to change direction along weaker interfaces [60].

Contrary to the study results, Rahman et al. [34] observed that Ribbond positioned at the base of the cavity achieved the best failure mode results, whereas placement in an occlusal groove resulted in the poorest outcomes. This contradiction could be due to differences in the study design as well as difference in the size of the fibers used.

The use of polyethylene fiber appears to be a promising technique when applied in an occlusal splinting direction. However, clinical trials should be carried out to confirm its beneficial performance in clinical settings. In vivo assessments of the clinical outcomes associated with each technique will aid in identifying the most effective method for fiber placement.

## 5. Conclusions

1. Strategic placement of long polyethylene fibers—particularly in an occlusal splinting configuration—can significantly enhance the fracture resistance of restored teeth, achieving performance levels comparable to those of sound teeth.
2. Placing fibers on the pulpal floor or in a circumferential orientation does not improve fracture resistance relative to conventional composite layering without reinforcement.
3. Both occlusal splinting and circumferential placement of fibers were associated with more restorable fracture patterns, highlighting the potential of these fiber reinforcement techniques to improve the clinical manageability of structurally compromised teeth.

## Figures and Tables

**Table 1:** One-way ANOVA demonstrating the statistically significant effect of different restoration treatments on the fracture resistance load.

Source	DF	Sum of Square	Mean Square	F Statistic	*p*-Value
Groups (between groups)	5	4,007,787.839	801,557.5678	12.0535	<0.0001
Error (within groups)	34	2,261,006.712	66,500.1974		
Total	39	6,268,794.551	160,738.3218		

DF, degrees of freedom; *p*-value, probability value.

**Table 2:** Fracture resistance values in Newtons (N) of tested groups.

Group	Descriptive Statistics				
	(n)	Minimum	Maximum	Mean	SD
Group 1	10	667.05	2008.41	1264.13 ^a^	559.74
Group 2	10	184.58	259.91	214.87 ^c^	34.49
Group 3	10	447.73	609.71	546.93 ^bc^	59.89
Group 4	10	415.21	919.27	621.08 ^bc^	171.61
Group 5	10	409.56	880.07	734.73 ^b^	154.79
Group 6	10	755.90	1687.46	1137.72 ^a^	316.20

n, sample number; SD, standard deviation. Different lower-case letters indicate a significant difference.

**Table 3:** Frequencies (n) and Percentages (%) for comparison between failure modes of different groups.

Group	Failure Mode				Significance
	Non-Restorable Failure		Restorable Failure		
	n *	%	n	%	
Sound	1	10	9	90	a
Cavity	8	80	2	20	b
Composite	6	60	4	40	bc
Pulpal Floor	5	50	5	50	ac
Wallpapering	3	30	7	70	a
Occlusal Groove	1	10	9	90	a

* n = 10, different lower-case letters indicate a significant difference.

## Data Availability

The raw data supporting the conclusions of this article will be made available by the corresponding author on request.

## Abbreviations

The following abbreviations are used in this manuscript:

CEJ	Cemento-enamel junction
ETT	Endodontically treated teeth
FRC	Fiber-reinforced composite
LWUHMW	Leno woven ultra-high molecular weight
MOD	Mesio-occluso-distal
SFRC	Short-fiber-reinforced composite

## References

[1] Sfeikos T., Dionysopoulos D., Kouros P., Naka O., Tolidis K. (2022). Effect of a fiber-reinforcing technique for direct composite restorations of structurally compromised teeth on marginal microleakage. J. Esthet. Restor. Dent..

[2] Albar N.H.M., Khayat W.F. (2022). Evaluation of Fracture Strength of Fiber-Reinforced Direct Composite Resin Restorations: An In Vitro Study. Polymers.

[3] Ilie N., Hilton T.J., Heintze S.D., Hickel R., Watts D.C., Silikas N., Stansbury J.W., Cadenaroi M., Ferracane J.L. (2017). Academy of Dental Materials guidance—Resin composites: Part I—Mechanical properties. Dent. Mater..

[4] Moosavi H., Zeynali M., Pour Z.H. (2012). Fracture resistance of premolars restored by various types and placement techniques of resin composites. Int. J. Dent..

[5] Demarco F.F., Corrêa M.B., Cenci M.S., Moraes R.R., Opdam N.J.M. (2012). Longevity of posterior composite restorations: Not only a matter of materials. Dent. Mater..

[6] Ástvaldsdóttir Á., Dagerhamn J., van Dijken J.W., Naimi-Akbar A., Sandborgh-Englund G., Tranæus S., Nilsson M. (2015). Longevity of posterior resin composite restorations in adults–A systematic review. J. Dent..

[7] Peumans M., Politano G., Van Meerbeek B., Leuven K.U. (2020). Effective Protocol for Daily High-quality Direct Posterior Composite Restorations. Cavity Preparation and Design. J. Adhes. Dent..

[8] Agrawal V., Shah A., Kapoor S. (2022). Effect of fiber orientation and placement on fracture resistance of large class II mesio-occluso-distal cavities in maxillary premolars: An in vitro study. J. Conserv. Dent..

[9] Scotti N., Michelotto Tempesta R., Pasqualini D., Baldi A., Vergano E.A., Baldissara P., Alovisi M., Comba A. (2020). 3D interfacial gap and fracture resistance of endodontically treated premolars restored with fiber-reinforced composites. J. Adhes. Dent..

[10] Zhang X., Zhang Q., Meng X., Ye Y., Feng D., Xue J., Wang H., Huang H., Wang M., Wang J. (2021). Rheological and mechanical properties of resin-based materials applied in dental restorations. Polymers.

[11] Jakab A., Volom A., Sáry T., Vincze-Bandi E., Braunitzer G., Alleman D., Garoushi S., Fráter M. (2022). Mechanical Performance of Direct Restorative Techniques Utilizing Long Fibers for “Horizontal Splinting” to Reinforce Deep MOD Cavities—An Updated Literature Review. Polymers.

[12] Mangoush E., Garoushi S., Lassila L., Vallittu P.K., Säilynoja E. (2021). Effect of fiber reinforcement type on the performance of large posterior restorations: A review of in vitro studies. Polymers.

[13] Butterworth C., Ellakwa A.E., Shortall A. (2003). Fibre-reinforced composites in restorative dentistry. Dent. Update.

[14] Avcılar İ.H., Bakır Ş. (2023). Use of fiber-containing materials in restorative dentistry. J. Dent. Sci. Educ..

[15] Rajak D.K., Pagar D.D., Menezes P.L., Linul E. (2019). Fiber-reinforced polymer composites: Manufacturing, properties, and applications. Polymers.

[16] Vallittu P., Özcan M. (2017). Clinical Guide to Principles of Fiber-Reinforced Composites in Dentistry.

[17] Dyer S.R., Lassila L.V.J., Jokinen M., Vallittu P.K. (2004). Effect of fiber position and orientation on fracture load of fiber-reinforced composite. Dent. Mater..

[18] Belli S., Erdemir A., Yildirim C. (2006). Reinforcement effect of polyethylene fibre in root-filled teeth: Comparison of two restoration techniques. Int. Endod. J..

[19] Belli S., Cobankara F.K., Eraslan O., Eskitascioglu G., Karbhari V. (2006). The effect of fiber insertion on fracture resistance of endodontically treated molars with MOD cavity and reattached fractured lingual cusps. J. Biomed. Mater. Res. B Appl. Biomater..

[20] Belli S., Erdemir A., Ozcopur M., Eskitascioglu G. (2005). The effect of fibre insertion on fracture resistance of root filled molar teeth with MOD preparations restored with composite. Int. Endod. J..

[21] Bijelic-Donova J., Bath A.K., Rocca G.T., Bella E.D., Saratti C.M. (2025). Can Fiber-reinforced Composites Increase the Fracture Resistance of Direct Composite Restorations in Structurally Compromised Teeth? A Systematic Review and Meta-analysis of Laboratory Studies. Oper. Dent..

[22] Ferrando Cascales Á., Andreu Murillo A., Ferrando Cascales R., Agustín-Panadero R., Sauro S., Carreras-Presas C.M., Hirata R., Lijnev A. (2025). Revolutionizing Restorative Dentistry: The Role of Polyethylene Fiber in Biomimetic Dentin Reinforcement—Insights from In Vitro Research. J. Funct. Biomater..

[23] Abdulamir S.W., Majeed M.A. (2023). Fracture resistance of endodontically treated maxillary premolar teeth restored with wallpapering technique: A comparative in vitro study. Int. J. Dent..

[24] Sáry T., Garoushi S., Braunitzer G., Alleman D., Volom A., Fráter M. (2019). Fracture behaviour of MOD restorations reinforced by various fibre-reinforced techniques—An in vitro study. J. Mech. Behav. Biomed. Mater..

[25] Taha N.A., Palamara J.E., Messer H.H. (2011). Fracture strength and fracture patterns of root filled teeth restored with direct resin restorations. J. Dent..

[26] Ramírez-Gómez J.F., Ortiz-Magdaleno M., Zavala-Alonso N.V. (2024). Effect of polyethylene fiber orientation on fracture resistance of endodontically treated premolars. J. Prosthet. Dent..

[27] Soto-Cadena S.L., Zavala-Alonso N.V., Cerda-Cristerna B.I., Ortiz-Magdaleno M. (2023). Effect of short fiber-reinforced composite combined with polyethylene fibers on fracture resistance of endodontically treated premolars. J. Prosthet. Dent..

[28] Singhal S., Gurtu A., Singhal A., Bansal R., Mohan S. (2017). Effect of different composite restorations on the cuspal deflection of premolars restored with different insertion techniques—An in vitro study. J. Clin. Diagn. Res..

[29] Forster A., Braunitzer G., Tóth M., Szabó B.P., Fráter M. (2019). In Vitro Fracture Resistance of Adhesively Restored Molar Teeth with Different MOD Cavity Dimensions. J. Prosthodont..

[30] Özüdoğru S., Tosun G. (2022). Evaluation of Microleakage and Fatigue Behaviour of Several Fiber Application Techniques in Composite Restorations. Ann. Dent. Spec..

[31] Deliperi S., Alleman D., Rudo D. (2017). Stress-reduced direct composites for the restoration of structurally compromised teeth: Fiber design according to the “wallpapering” technique. Oper. Dent..

[32] Khan S.I.R., Ramachandran A., Alfadley A., Baskaradoss J.K. (2018). Ex vivo fracture resistance of teeth restored with glass and fiber reinforced composite resin. J. Mech. Behav. Biomed. Mater..

[33] Akman S., Akman M., Eskitascioglu G., Belli S. (2011). Influence of several fibre-reinforced composite restoration techniques on cusp movement and fracture strength of molar teeth. Int. Endod. J..

[34] Rahman H., Singh S., Chandra A., Chandra R., Tripathi S. (2016). Evaluation of fracture resistance of endodontically treated teeth restored with composite resin along with fibre insertion in different positions in vitro. Aust. Endod. J..

[35] Hamouda I.M., Shehata S.H. (2011). Fracture resistance of posterior teeth restored with modern restorative materials. J. Biomed. Res..

[36] Mergulhão V.A., De Mendonça L.S., De Albuquerque M.S., Braz R. (2019). Fracture resistance of endodontically treated maxillary premolars restored with different methods. Oper. Dent..

[37] Megahed M.S., Zaghloul A.I. (2020). Fracture Resistance of Maxillary Premolar Teeth Restored with Bulk Fill Resin Composite: In-vitro Study. Mansoura J. Dent..

[38] Gale M.S., Darvell B.W. (1999). Thermal cycling procedures for laboratory testing of dental restorations. J. Dent..

[39] Morresi A.L., D’Amario M., Capogreco M., Gatto R., Marzo G., D’Arcangelo C., Monaco A. (2014). Thermal cycling for restorative materials: Does a standardized protocol exist in laboratory testing? A literature review. J. Mech. Behav. Biomed. Mater..

[40] Taher H.M., Haridy M. (2019). Fracture resistance of maxillary premolars restored with different fiber-reinforced composites: An in vitro study. Egypt. Dent. J..

[41] Soares C.J., Pizi E.C., Fonseca R.B., Martins L.R. (2005). Influence of root embedment material and periodontal ligament simulation on fracture resistance tests. Braz. Oral. Res..

[42] Mincik J., Urban D., Timkova S., Urban R. (2016). Fracture resistance of endodontically treated maxillary premolars restored by various direct filling materials: An in vitro study. Int. J. Biomater..

[43] Battancs E., Sáry T., Molnár J., Braunitzer G., Skolnikovics M., Schindler Á., Szabó P. B., Garoushi S., Fráter M. (2022). Fracture Resistance and Microleakage around Direct Restorations in High C-Factor Cavities. Polymers.

[44] Fráter M., Sáry T., Vincze-Bandi E., Volom A., Braunitzer G., Szabó P. B., Garoushi S., Forster A. (2021). Fracture behavior of short fiber-reinforced direct restorations in large MOD cavities. Polymers.

[45] Scotti N., Coero Borga F.A., Alovisi M., Rota R., Pasqualini D., Berutti E. (2012). Is fracture resistance of endodontically treated mandibular molars restored with indirect onlay composite restorations influenced by fibre post insertion?. J. Dent..

[46] Szabó P.B., Sáry T., Szabó B. (2019). The key elements of conducting load-to-fracture mechanical testing on restoration-tooth units in restorative dentistry. Analecta Tech. Szeged..

[47] Singer L., Fouda A., Bourauel C. (2023). Biomimetic approaches and materials in restorative and regenerative dentistry. BMC Oral Health.

[48] Sengun A., Cobankara F.K., Orucoglu H. (2008). Effect of a new restoration technique on fracture resistance of endodontically treated teeth. Dent. Traumatol..

[49] Szabó V.T., Szabó B., Barcsayné-Tátrai N., Mészáros C., Braunitzer G., Szabó B.P., Lassila L., Garoushi S., Fráter M. (2023). Fatigue Resistance of Dissected Lower First Molars Restored with Direct Fiber-Reinforced Bridges—An In Vitro Pilot Study. Polymers.

[50] Khan S., Sitlani M., Pandey S., Singh S.K., Mishra P., Narang A. (2023). To Study Fracture Resistance of Interlig^TM^ Glass Fiber Orientation and Placement on Large Class II Cavities in Maxillary Premolars: An in Vitro Study. Dent. J..

[51] Hegde V., Sali A.V. (2017). Fracture resistance of posterior teeth restored with high-viscosity bulk-fill resin composites in comparison to the incremental placement technique. J. Conserv. Dent..

[52] Hood J.A. (1991). Biomechanics of the intact, prepared and restored tooth: Some clinical implications. Int. Dent. J..

[53] Ayad M.F., Maghrabi A.A., Garcia-Godoy F. (2010). Resin composite polyethylene fiber reinforcement: Effect on fracture resistance of weakened marginal ridges. Am. J. Dent..

[54] Hazar E., Hazar A. (2024). Effect of Long Glass Fiber Orientations or a Short-Fiber-Reinforced Composite on the Fracture Resistance of Endodontically Treated Premolars. Polymers.

[55] Oskoee P.A., Ajami A.A., Navimipour E.J., Oskoee S.S., Sadjadi J. (2009). The Effect of Three Composite Fiber Insertion Techniques on Fracture Resistance of Root-filled Teeth. J. Endod..

[56] Küçük Ö., Keçeci A.D. (2021). Strengthening effect of different fiber placement designs on root canal treated and bleached premolars. Odontology.

[57] Bahari M., Mohammadi N., Kimyai S., Kahnamoui M.A., Vahedpour H., Torkani M.A.M., Oskoee A.S. (2019). Effect of Different Fiber Reinforcement Strategies on the Fracture Strength of Composite Resin Restored Endodontically Treated Premolars. Pesqui. Bras. Odontopediatria Clin. Integr..

[58] Fráter M., Lassila L., Braunitzer G., Vallittu P.K., Garoushi S. (2020). Fracture resistance and marginal gap formation of post-core restorations: Influence of different fiber-reinforced composites. Clin. Oral Investig..

[59] Lee J.J.W., Kwon J.Y., Chai H., Lucas P.W., Thompson V.P., Lawn B.R. (2009). Fracture modes in human teeth. J. Dent. Res..

[60] Belli S.E., Eskitascioglu G.Ü. (2006). Biomechanical properties and clinical use of a polyethylene fibre post-core material. Int. Dent. S. Afr..

